# Self-Healing Hydrogels with both LCST and UCST through Cross-Linking Induced Thermo-Response

**DOI:** 10.3390/polym11030490

**Published:** 2019-03-13

**Authors:** Haifeng Zhao, Heng An, Baozhong Xi, Yan Yang, Jianglei Qin, Yong Wang, Yingna He, Xinguo Wang

**Affiliations:** 1 Hebei Key Laboratory of Chinese Medicine Research on Cardio-Cerebrovascular Disease, Pharmaceutical College, Hebei University of Chinese Medicine, Shijiazhuang 050200, China; Zhaohf226@126.com (H.Z.); xibaoz567@163.com (B.X.); 15176821860@163.com (Y.Y.); 2 College of Chemistry and Environmental Science, Hebei University, Baoding 071002, China; flyingstock@foxmail.com (H.A.); qinhbu@iccas.ac.cn (J.Q.); 3 Medical College, Hebei University, Baoding 071002, China; ywangnk@163.com; 4 Hebei TCM Formula Granule Technology Innovation Center & TCM Formula Granule Research Center of Hebei Province University, Hebei University of Chinese Medicine, Shijiazhuang 050200, China

**Keywords:** P(AM-*stat*-DAA), self-healing hydrogel, cross-linking induced thermo-response

## Abstract

Self-healing hydrogels have drawngreat attention in the past decade since the self-healing property is one of the characteristics of living creatures. In this study, poly(acrylamide-stat-diacetone acrylamide) P(AM-*stat*-DAA) with a pendant ketone group was synthesized from easy accessible monomers, and thermo-responsive self-healing hydrogels were prepared through a series of diacylhydrazide compounds cross-linking without any additional stimulus. Although the copolymers do not show thermo-response, the hydrogels became thermo-responsive andboth the lower critical solution temperature (LCST) and upper critical solution temperature (UCST) varied with the composition of the copolymer and structure of cross-linkers. With a dynamic covalent bond connection, the hydrogel showed gel-sol-gel transition triggered by acidity, redox, and ketone to acylhydrazide group ratios. This is another interesting cross-linking induced thermo-responsive (CIT) hydrogel with different properties compared to PNIPAM-based thermo-responsive hydrogels. The self-healing hydrogel with CIT properties could have great potential for application in areas related to bioscience, life simulation, and temperature switching.

## 1. Introduction

Hydrogels are an appealing class of materials for applications in biomedical and bio-engineering fields, and offer a number of functional benefits as a result of their high water content and solid-like mechanical properties [1,2]. However, one of the disadvantages of using conventional hydrogels inbiological systems is their ease of damage or fatigue duringnormal operation, limiting theirlifetime [3]. Therefore, designing mechanically robust hydrogels with a self-healing capabilityishighly desirable for effectively increasingthe lifespan and prolongingthe durability and reliability of the hydrogels.

Self-healing hydrogels can automatically heal damage and restorethemselves to normality without the intervention of external stimuli, whichis similar to some living organisms. Self-healing hydrogelswith excellent biocompatibility have been developed as a promising and successful material system for many biomedical applications, including biosensors [4], controlled drug delivery [5,6], wound healing [7], etc.

In the past few years, scientists have designed a variety of smart self-healing materials based on the intermolecular force [8,9,10] and dynamic covalent bonds [11]. Compared to self-healingmaterials based on host-guest interactions [12] and H-bonds with fast self-healing [9,13], the dynamic covalent bonds always endow the materials with a higher dimension stability, better mechanical properties, and solvent resistance [14,15,16]. Therefore, alarge amount of self-healing polymer materials have beenprepared through reversible covalent bonds of the Diels-Alder reaction [17], diarylbibenzofuranone [18,19], boronic ester [20,21], Schiff-base [15,22,23], disulfide bonds [24], etc. in the past decades and have also made great progress [25,26,27,28].

The boronic ester bond was used to prepare a variety of self-healing hydrogels [29,30,31,32]. Sumerlin’s group reported an oxime bond-based self-healing hydrogelfrom P(DMA-stat-DAA) [33]. Other kinds of Schiff-base have beenmore widely used to prepare self-healing hydrogels. The hydrogel can be prepared from chitosan and carbonyl group containing compounds [28,34,35]. However, the acylhydrazone bond-based self-healing hydrogel ismore popular since the acylhydrazide can be conveniently transformed from an ester bond or carboxylic acid [27,36,37,38] and the acylhydrazone bond-based hydrogels can form and self-heal under neutral conditions, without any stimulus [38,39].

Besides self-healing, smart hydrogels with thermo-response around body temperature are especially attractive since the properties do not require direct contact, resulting ina minimal impact on materials, and can be easily controlled [40]. However most thermo-responsive hydrogels are based on poly(*N*-isopropylacrylamide)(PNIPAM) and its copolymers due totheir LCST in water up to now, and the phase transition temperature is limited to acertain range and hard to manipulate precisely [28,41,42]. In our recent research, thermo-responsive hydrogels were prepared from non-thermo-responsive P(DMA-stat-DAA), and the cross-linked structure regulated the cloud point (CP) of the hydrogels and gavethem the ability of thermo-response. Because the thermo-response was generated by cross-linking, this property was named the cross-linking induced thermo-response (CIT) [43,44]. The CIT property opened a new window to prepare thermo-responsive hydrogels with materials in a wider phase transition temperature range, although related research was not investigated intensively.Our recent research has also revealed that self-healing hydrogels prepared from P(AM-*stat*-DAA) showed the transition of a clear hydrogel to an opaque hydrogel with increasing DAA composition, and this phenomenon was very similar to P(DMA-stat-DAA), which indicated that this kind of copolymer could also exhibit aCIT property [45].

**Scheme 1:**
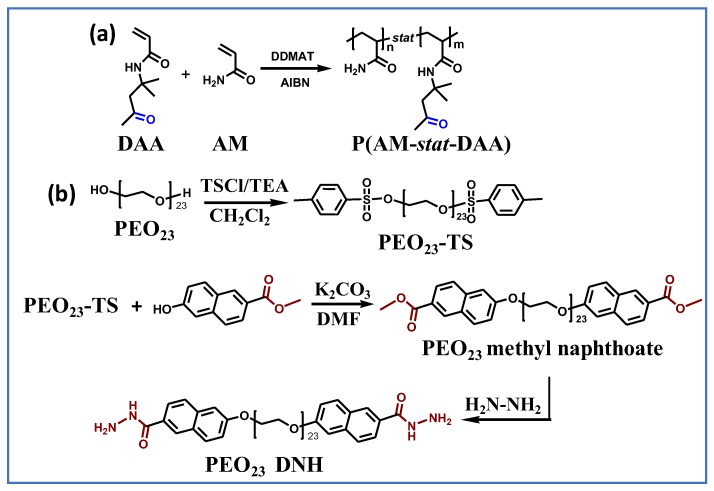
Synthesis of P(AM-*stat*-DAA) through RAFT polymerization (**a**) and synthesis of PEO_23_ DNH (**b**).

In this research, self-healing hydrogels with a CIT propertywere prepared from P(AM-*stat*-DAA) with diacylhydrazide cross-linking. It was demonstrated thatthe hydrogel could form and self-heal without any external stimulus and showed both UCST and LCST with different diacylhydrazide as cross-linkers. The hydrogel with PEO_23_ dinaphthoylacylhydrazide (PEO_23_ DNH) showed LCST around body temperature, while ADH and DTDPH cross-linked hydrogel showed UCST. The synthesis of P(AM-*stat*-DAA)and PEO23 DNHisshown in Scheme 1. At the same time, the hydrogels showed reversible gel-sol-gel transition by multi-stimulus. Compared to thermo-responsive hydrogels based on PNIPAM, the hydrogels with a CIT property [43] inspired more possibility to design thermo-sensitive hydrogels with easy regulation [37,46]. Moreover, the CIT property provides abetter application property in areas related to bioscience and biotechnology and more possibility to mimic living creatures at different temperatures.

## 2. Experimental section

### 2.1. Materials

Diacetone acrylamide (DAA) and 6-hydroxy-2-naphthoic acid were purchased from Macklin Co. Acrylamide (AM) was supplied by Tianjin Damao Chemical Co. (Tianjin, China). The DAA and AM were recrystallized in *n*-hexane three timesto remove the inhibitor before copolymerization. Dimethyl 3,3′-dithiodipropionateand adipicdihydrazide (ADH) were also purchased from Macklin Co. Ltd. (Shanghai, China). Dithiodipropionic acid dihydrazide (DTDPH) was prepared from dimethyl 3,3′-dithiodipropionate through hydrazinolysis [36,37]. Poly(ethylene oxide) (PEO, DP = 23) was supplied by the Guangfu fine chemical research institute (Tianjin, China) and used to prepare PEO_23_ dibenzoylacylhydrazide (PEO DBH) and PEO_23_ dinaphthoylacylhydrazide (PEO DNH), according to a previous report [44]. The RAFT agent S-1-Dodecyl-S′-(R,R′-dimethyl-R″-acetic acid) trithiocarbonate (DDMAT) was synthesized in our lab [47]. Azodiisobutyronitrile (AIBN), dimethylforamide (DMF), methanol, dithiothreitol (DTT), H_2_O_2_, HCl, triethylamine (TEA), and all other solvents and reagents were supplied by Kermel Chemical Reagent Co. ( Tianjin, China) and used as received.

### 2.2. Characterizations

The compositions of copolymers were determined by ^1^H NMR spectra, which were obtained on a Bruker 600 MHz spectrometer (Avance III, Bruker Co. Karlsruhe, Germany) in D_2_O at room temperature. The Fourier-transform infrared (FT-IR) spectra were obtained on a Varian 600 IR spectrometer. The transmittances of the polymer solution and hydrogels were characterized on a Shimadzu UV-2550 UV-Vis spectrophotometer equipped with an automatic temperature control accessory, and the absorbance at a 500 nm wavelength was recorded to determine the phase transition. A Scanning Electron Microscopy (SEM) image was observed on a JSM-7500 microscope to characterize the morphology of the hydrogels after lyophillization with the operating voltage at 10 kV. A differential scanning calorimeter (Diamond DSC, Perkin Elmer, Waltham, MA, USA) was used to determine the *T*_g_ of the copolymers under N_2_ atmosphere at a heating rate of 20 °C min^−1^. Thermal stability of the lyophillized hydrogels was characterized by thermogravimetric analysis (TGA, Pyris 1, Perkin Elmer), and the samples were heated from room temperature to 800 °C at a heating rate 20 °C min^−1^ under N_2_ atmosphere.

### 2.3. Polymerization of AM and DAA Mediated by DDMAT

RAFT polymerization was applied to prepare P(AM-*stat*-DAA) with various compositions, the DDMAT was used to mediate the polymerization initiated by AIBN, the mechanism isillustrated in Scheme 1a, and the experiment was carried out with the following procedure: [43,44,45] DDMAT (145.6 mg, 0.4 mmol), AM (2.84 g, 40 mmol), DAA (1.35 g, 8 mmol), and AIBN (9.8 mg, 0.12 mmol) were dissolved in 10 mL DMF in a 20 mL reaction tube equipped with a magnetic stirring bar, the tube was then sealed, and the oxygen was removed by three freeze-pump-thaw cycles. After being filled with nitrogen, the mixture in the reaction tube was melted and the tube was immersed into a 60 °C oil bath. The polymerization was performed for 24 h under N_2_ atmosphere with continuous stirring. The polymer product of P(AM-*stat*-DAA) was precipitated in methanol three times and dries under vacuum. The polymer was then dissolved in water and lyophilized to collect the product. The molecular weight of the polymer was evaluated by monomer conversion and the composition of the polymer was confirmed based on the ^1^H NMR spectrum.

### 2.4. Synthesis of PEO_23_DNH

The preparation of PEO DNH was carried out following the same procedure asPEO DBH with the methyl 4-hydroxybenzoate changed to methyl 6-hydroxy-2-methyl naphthoate [43], as shown in Scheme 1b. First, the 6-hydroxy-2-naphthoic acid was reacted with methanol to prepare 2-hydroxy-6-methyl naphthoate. Then, PEO_23_ toluene sulfonate (PEO_23_-TS) was dissolved in DMF, and6-hydroxy-2-methyl naphthoate and K_2_CO_3_ were added into the solution. After being stirred for 72 h under 80 °C, the solution was diluted with water and extracted threetimes with CH_2_Cl_2_. The organic phase was washed by Na_2_CO_3_ solution threetimes and dried by anhydrous MgSO_4_. Then, the solution was filtrated, concentrated, and precipitated in petroleum/ethyl acetate (8/2) twice to obtain PEO_23_ methyl naphthoate. The PEO_23_ methyl naphthoate was dissolved in methanol, hydrazine (80%) was added three times, and the reaction was performed for 72 h under N_2_. The solution was diluted withwater and extracted by CH_2_Cl_2_. The organic phase was dried by MgSO_4_ and precipitated in petroleum/ethyl acetate (8/2*V*/*V*). Then, the product was dissolved in water and lyophilized to obtain PEO_23_ DNH.

### 2.5. Preparation of Self-Healing Hydrogel Containing Ketone type Acylhydrazone Bonds

The P(AM-*stat*-DAA) with various compositions wasused to prepare ketone type acylhydrazone cross-linked hydrogels with a variety of diacylhydrazide compounds as cross-linkers through the following procedure: First, P(AM-*stat*-DAA) and a stoichiometric of thedihydrazide compound were dissolved in deionized water with a total concentration of 10%, and then the solution was put into the corresponding molds or disposable PS cuvettes to form hydrogel without any interference. Hydrogel formation was confirmed by vial leaning. A rheology test and self-healing experimentwere conductedafter the hydrogel was formed and incubated for 24 h to reach the equilibrium state.

The rheological test of the hydrogels was conducted on a TA AR2000ex rheometer(TA Instruments, USA) under oscillatory mode. A25 mm parallel plate was used in the experiment and the gap was fixed at 1000 μm for all measurements. The frequency was scanned from 0.05–100 rad s^−1^ with the strain of 5% within the linear viscoelastic regime. The strain-controlled mode was carried out at 1 rad s^−1^.

The self-healing experiment of the hydrogels was carried out according to the following procedures: The hydrogels with various compositions and cross-linkers were incubated for 24 h before study. Then, the hydrogels were cut into halves across the center, and hydrogel pieces were put back into the original mould with the cut surface contact closely. The healing result was confirmed by stretching the healed hydrogel with tweezers from both sides or subjecting to gravity after 24 h. Photos were taken at each step with a digital camera to track the processes.

### 2.6. Thermo-Response of Hydrogels Regulated by Composition and Cross-linkers

The thermo-response of the copolymer and the hydrogels were characterized by determining the transmittance of the solution and the hydrogels using a temperature controlled UV-Vis spectrophotometer. The change in transmittance with increasing temperature was recorded at a 500 nm wavelength. The phase transition temperature was defined as the temperature when the transmittance increased or decreased to the mid-value before and after the phase transition.

### 2.7. Gel-Sol-Gel Transition of the Hydrogels Under a Variety of Triggers

As a typical reversible covalent bond, the acylhydrazone bond can go reversibly when acid is added to consume acylhydrazide. As a result, the hydrogel de-cross-linked and turned into liquid [27]. To investigate the pH triggered gel-sol-gel transition of this hydrogel, HCl was added into the hydrogel vial to regulate the pH of the hydrogel to about 3. The gel-sol transition of the hydrogel was observed by leaning the vial and recorded by a digital camera. Then, triethylamine was added to neutralize the acid and regulate the pH of the solution to 6. The sol-gel transition process was also recorded by the digital camera.

The hydrogel prepared with DTDPH as the cross-linker contains a redox sensitive disulfide bond connection, which can be reduced into a thiol group by DTT, *n*-Bu_3_P [48], etc. In this research, the redox triggered gel-sol-gel transition of the hydrogels with DTDPH cross-linking wasinvestigated. The DTT (two times based on the disulfide bond) was added into the DTDPH cross-linked hydrogel to reduce the disulfide bond. After the hydrogel was degraded into liquid, the same amount of H_2_O_2_ as the oxidant was added to the solution to see if the disulfide bond couldbe reformed to obtain hydrogel again.

The acylhydrazone exchange reaction in the self-healing hydrogel was also in progress in the hydrogel itself, so the hydrogel couldbe cleaved by a monofunctional compound [33]. In this research, diacylhydrazide cross-linkers were used to investigate the group ratio triggered gel-sol-gel transition of the hydrogels [43,49]. Three equivalent amounts of diacylhydrazinewereadded into the hydrogel prepared from P(AM-*stat*-DAA) to regulate the group ratios of ketone group:acylhydrazide to 1:4. The cleavage of the hydrogel into liquid was observed and recorded by a digital camera. Then, P(AM-*stat*-DAA) solution was added to regulate the group ratio to 1:1 to observe the re-formation of the hydrogel.

### 2.8. Morphology Observation of the Self-Healing Hydrogels with Different Cross-linker and Transparency

The structures of the hydrogels with different cross-linkers were determined by SEM observation. The hydrogels prepared from P(AM-*stat*-ADAA) with various cross-linkers and different transparencies were observed. The hydrogels were frozen in liquid N_2_ to avoid phase transition. Then, the frozen hydrogels were lyophilized and coated with Au for SEM observation. The SEM images were observed on a JSM-7500 microscope (JEOL, Osaka, Japan) with an operating voltage of 10 kV and the structures of the hydrogel were compared based on the images.

## 3. Results and Discussion

### 3.1. Preparation of P(AM-*stat*-DAA) with Pendant Ketone Groups and Synthesis of PEO_23_ DNH

**Figure 1:**
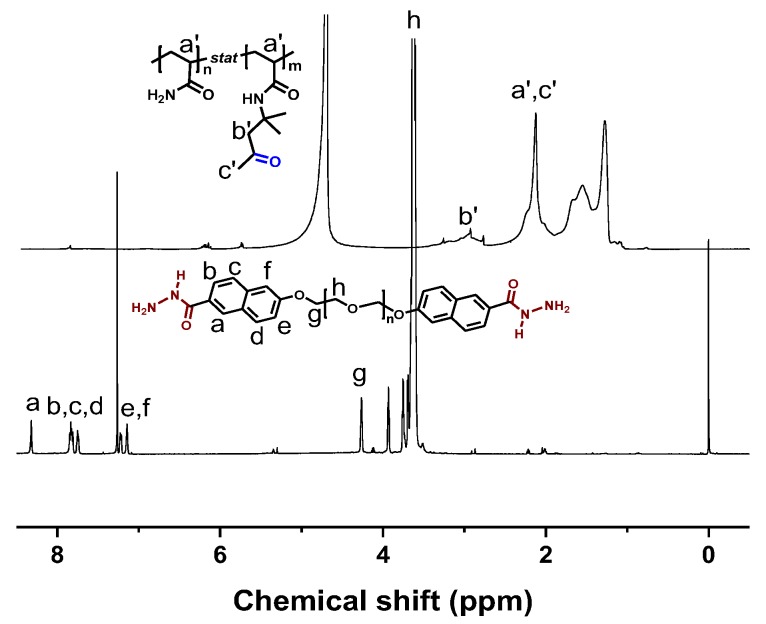
^1^H NMR spectrum of P(AM-*stat*-DAA_20_) (**top**) and PEO_23_ DNH (**bottom**) in CDCl_3_.

RAFT polymerization is widely used to prepared polymers with well-controlled polydispersity and was used to prepare the P(AM-*stat*-DAA) copolymers with various ketone functional densities (Scheme 1a) [43,45]. The copolymer containing ketone group can be cross-linked to form dynamic oxime bond- or acylhydrazone bond-based self-healing hydrogels, and theP(DMA-*stat*-DAA)-based self-healing hydrogel showed that cross-linking induced thermo-responsiveness [33]. The molecular weights (*M*_n_) of the P(AM-*stat*-DAA) copolymers were evaluated by monomer conversion. The composition of the copolymers was almost consistent, with the monomer feeding ratio being characterized by comparing the peak areas of ^1^H NMR spectra [45]. The ^1^H NMR spectrum of the P(AM-*stat*-DAA) is shown in Figure 1 (top) as an example. To investigate the influence of composition on properties of the hydrogel, four copolymer samples with increasing DAA compositions were synthesized and are listed in Table 1.

The PEO_23_ DNH was synthesized through the hydrazinolysis of PEO_23_ methyl naphthoatesimilar to the synthesis of the PEO_23_ DBH. After a three-step reaction, brown colored PEO_23_ DNH was obtained because of the naphthalene moiety. The structure of PEO_23_ DNH was characterized by ^1^H NMR, as shown in Figure 1 (bottom). The peaks from 8.38 ppm to 7.08 ppm (a–f) are derived from the naphthalene ring, while the g (4.26 ppm) and h (3.64 ppm) come from the PEO chain, and by comparing the peak areas of the corresponding protons, the functional ratio of the naphthoylhydrazide was found to be about 93% and was ready to be used to prepare hydrogel with an acylhydrazone connection.

**Figure 2:**
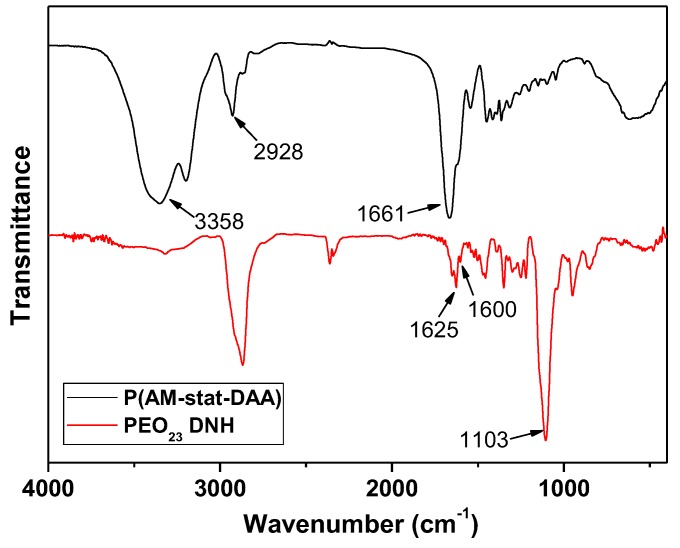
FT-IR spectra of P(AM_72_-*stat*-DAA_28_) (**top**) and PE_O23_ DNH (**bottom**).

The structures of the copolymers P(AM-*stat*-DAA) and PEO_23_ DNH were also characterized by FT-IR. One strong absorbance representing the amide carbonyl group was illustrated at 1661 cm^−1^ on the FT-IR spectrum of P(AM_72_-*stat*-DAA_28_), as shown in Figure 2 (top). However, the ketone group could notbe identified on the FT-IR spectrum because the absorbance was shielded, which was always located at 1710 cm^−1^ [44]. The FT-IR spectra of PEO_23_ DNH arealso illustrated in Figure 2. The absorbance of the carbonyl group appeared at 1625 cm^−1^, proving that the ester group was cleaved during hydrazinolysis to form the acylhydrazide group. The *T*_g_ of the copolymers was determined by DSC characterization. The result showed that the *T*_g_values of the copolymer were pretty high, with the *T*_g_of P1 being 188.8 °C, and the *T*_g_ value decreased with the increasing of DAA composition (Figure S1). This result indicated that although the DAA has a higher steric resistance based on its size, the H-bond force between AM segments reduced after copolymerization. However, the *T*_g_ of the copolymers was still pretty high, with all values beinghigher than 150 °C, as listed in Table 1. This decreasing of *T*_g_ also suggests that the DAA has copolymerized randomly with DMA and the ketone functional group has been imported onto the polymer chain.

### 3.2. Preparation of Hydrogels from P(AM-stat-DAA) Cross-Linked by Various Diacylhydrazides

**Figure 3:**
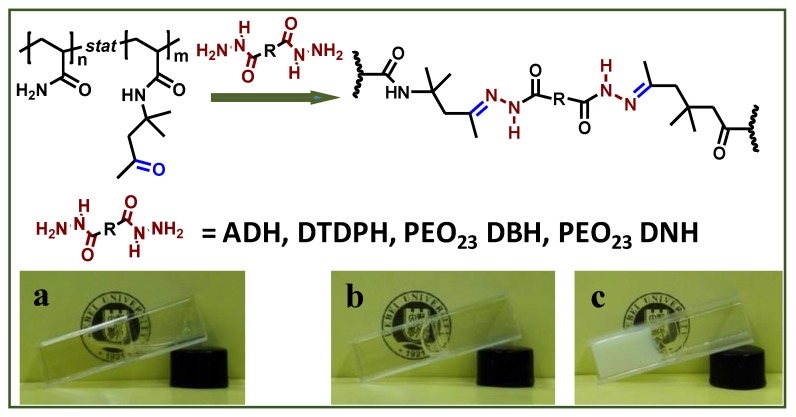
Hydrogels from P(AM-*stat*-DAA) and a variety of dihydrazide compounds (Scheme), and optical images of solution before and after gelation (**a**) before gelation; (**b**) clear hydrogel; (**c**) opaque hydrogel).

The P(AM-*stat*-DAA) sampleswith various compositions of ketone groups were used to prepare hydrogel with dynamic acylhydrazone bonds, as shown in Figure 3 (Scheme). The diacylhydrazide compounds of ADH, DTDPH, PEO_23_ DBH, and PEO_23_ DNH (Scheme S1) were used to cross-link the P(AM-*stat*-DAA) with different cross-linking densities. All the group ratios of ketone to acylhydrazide were fixed at 1:1. It was noticed that the gelation reaction time of this study was pretty long, and up to several hours without stimulus [45]. It was noticed that gelator solution transformed into clear hydrogels with PEO_23_ DBH, no matter the composition of the P(AM-*stat*-DAA), as shown in Figure 3a,b. The PEO_23_ DNH cross-linked hydrogels were brown and transparent because of the naphthalene ring. However, when the cross-linker changed to ADH, clear hydrogels formed fromP(AM_77_-*stat*-DAA_8_) and P(AM_71_-*stat*-DAA_14_), while hydrogel prepared from P(AM_65_-*stat*-DAA_20_) and P(AM_72_-*stat*-DAA_28_) become opaque. When the DTDPH was used to cross-link the P(AM-*stat*-DAA) to prepare hydrogels, clear hydrogel was obtained from polymer P(AM_77_-*stat*-DAA_8_) and opaque hydrogels were prepared from other copolymers, and the photographs of opaque hydrogels prepared from P(AM_82_-*stat*-DAA_16_) with DTDPH cross-linking areshown in Figure 3c.

### 3.3. Rheological Properties of Self-Healing Hydrogels

**Figure 4:**
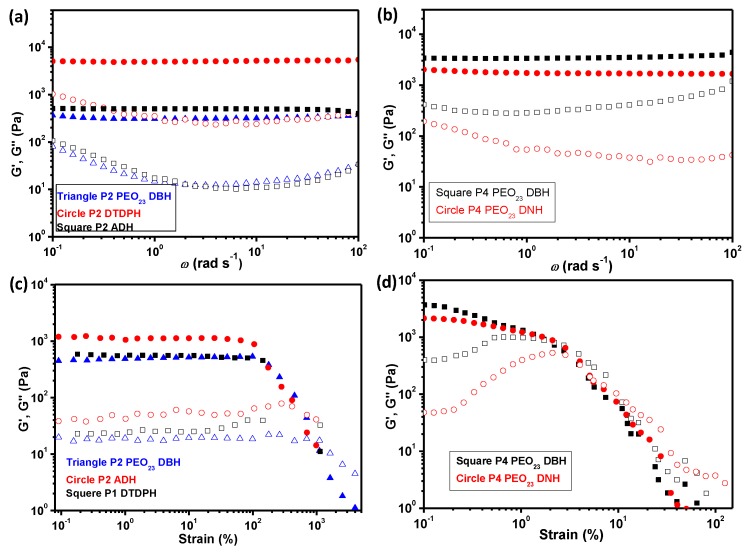
Rheology curves of a series of hydrogels prepared from various copolymers and cross-linkers at 25 °C. (**a**,**b**) frequency scan at 5% strain; (**c**,**d**) strain scan at 1 rad s^−1^. Solid: *G*′; Hollow: *G*″).

Besides vial leaning, the rheological study was also used to confirm the formation and compare the mechanical properties of the hydrogels [27,50]. All the hydrogels showed a solid characteristic with *G*′ > *G*″ in this research. The dependence of storage modulus (*G*′) and loss modulus (*G*″) of a series of hydrogels prepared from P(AM_71_-*stat*-DAA_14_) and P(AM_65_-*stat*-DAA_120_) on frequency were determined at 25 °C, and the results are shown in Figure 4a,b. The *G*′values of the hydrogels are higher than *G*″ with the frequency as low as 0.1 rad s^−1^, which indicatedsolid rheology characteristics of the hydrogels. The *G*′ vales of the clear hydrogels prepared from P(AM_71_-*stat*-DAA_14_) with ADH and PEO_23_ DBH cross-linking were lower than those for the DTDPH cross-linked one, and the reason for this was the DTDPH cross-linked hydrogel became opaque with hydrophobic domains aggregated and strengthened the hydrogel. Additionally, because the hydrophobic domains formed, this hydrogel becomes brittle at room temperature [45]. The *G*′ values of all the hydrogels are almost independent offrequency (solid symbols), but the *G*″ of the hydrogels increased gradually at a low frequency, indicating reversible characteristics of the acylhydrazone bond, which can endow the hydrogels with a self-healing property. With an increasing cross-linking density, the hydrogel prepared from polymer 4 with PEO_23_ DBH and PEO_23_ DNH showed higher *G*′ compared to polymer 2 (Figure 4b). The higher G′ always mean a higher strength of the hydrogels, so the clear hydrogels in this study provided a moderate strength that couldhold their own weight without distortion when subjected to gravity [50].

As the clear hydrogels were flexible, the flexibility of a series of hydrogels wascharacterized by strain amplitude sweeps at a frequency of 1 Hz. As exhibited in Figure 4c and d, the *G*′ of the hydrogels prepared from polymer 1 and polymer 2 presented a plateau at low strain and remainedconstant with the increasing of strain up to 100% strain; the *G*″ showed the same trend in the same strain range. When the strain further increased, the *G*′ dropped drastically to below the *G*″ and showed a liquid characteristic. Thisindicated fracture of the hydrogels. The clear hydrogels can sustain a 100% strain without breaking, and this property endows the hydrogels with wide areas of application without worrying about its brittleness. However, the transparent hydrogels prepared from polymer 4 showed a lower flexibility, which fractured below a 10% strain (Figure 4d).

### 3.4. Thermo-Responsive Property of Hydrazone Bond Containing Hydrogels

**Figure 5:**
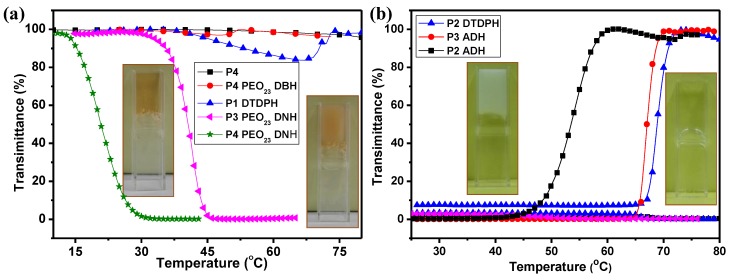
Transmittances of copolymers and hydrogels prepared from various copolymers and cross-linkers with increasing temperature. (**a**) LCST property; (**b**) UCST property. The inserted images are hydrogel below and above phase transition.

The hydrogels with different diacylhydrazide cross-linking turned from transparent to opaque with increasing DAA composition, and this phenomenon is very similar to those cross-linking-induced thermo-responsive (CIT) self-healing hydrogels prepared from P(DMA-*stat*-DAA) copolymers [43,44]. Therefore, the CIT property was expected and the thermo-responsive property of this system was studied. The transmittance vs. heating curves of a series of clear hydrogels at a 500 nm wavelength are illustrated in Figure 5. However, the clear hydrogels prepared from water soluble P(AM-*stat*-DAA) with ADH did not show any thermo-responsiveness, similarto PEO_23_ DBH cross-linked hydrogels and the copolymers (Figure 5a). The transmittance of the hydrogel prepared from P(AM_77_-*stat*-DAA_8_) and DTDPH decreased a little bit from 45 to 70 °C, indicating aggregation of the hydrophobic moiety. However, because the transparency onlydecreased by 20%, the hydrogel did not turn opaque at a high temperature. To regulate the hydrophilicity of the hydrogel, PEO_23_ DNH was prepared and used to cross-link the copolymer. When the cross-linker was changed into PEO_23_ DNH, the brown hydrogel that formed from P(AM_65_-*stat*-DAA_20_) showed thermo-response with an LCST of 40.6 °C. When the DAA composition further increased to P(AM_72_-*stat*-DAA_28_), the LCST further decreased to 20.9 °C (Figure 5a purple and green line), and the optical images of this hydrogel below and above phase transition are inserted into Figure 5a for comparison. This result indicated that a larger hydrophobic moiety was formed and regulated the thermo-response of the hydrogel. The thermo-responsive hydrogel from P(AM-*stat*-DAA) is very interesting since no any previous publication has reported the thermo-responsive property of PAM-based hydrogels [51]. The phase transition temperature is 40.6 °C, a little higher above body temperature, which could have great potential application in bio-science and tissue engineering. Moreover, the phase transition process wascompletely reversible, when the temperature decreased gradually, the transparency increased gradually, and the hydrogel became clear again (Figure S2).

**Scheme 2:**
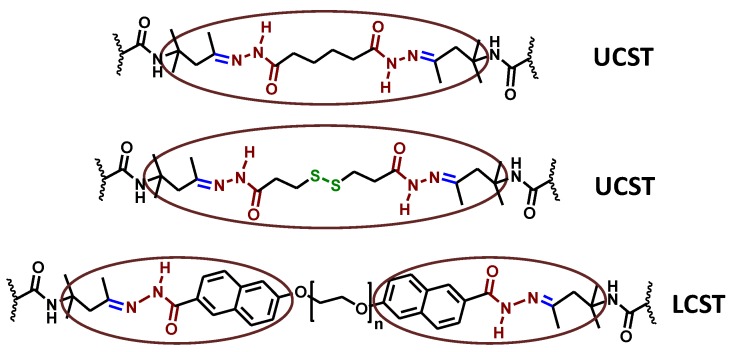
The hydrophobic moieties formed during cross-linking attributed to the thermo-responsiveness of the hydrogels.

Although the clear hydrogel with ADH cross-linking did not turn opaque upon heating, it was noticed that the hydrogel prepared from P(AM_71_-*stat*-DAA_14_) turned opaque slowly when cooled in a refrigerator (4 °C). This means that the hydrogels showed a thermo-response of UCST. Based on this property, the transmittance dependent on temperature of a series of opaque hydrogels wascharacterized, and the results are shown in Figure 5b. Although the hydrogel prepared from P(AM_71-_*stat*-DAA_14_) with ADH cross-linkingwasclear at room temperature, the phase transition temperature was 53.5 °C under transmittance determination (Figure 5b black line) and the hydrogels below and above UCST have beeninserted for comparison. If the hydrogel was warmed at 25 °C, about 10 h was needed to turn it from opaque to transparent, whichis because the melting of aggregated hydrophobic domains is very slow around UCST. When the composition of the copolymer changed to P(AM_65_-*stat*-DAA_20_), the UCST increased to 67.0 °C, while the P(AM_71_-*stat*-DAA_14_) with DTDPH cross-linking showed the UCST of 68.7 °C (Figure 5b red and blue lines). Other opaque hydrogels did not turn clear in our temperature range because of the high composition of the hydrophobic segment. This result is very interesting since no report has shownthermo-response of the P(AM-*stat*-DAA) copolymers with the design ofahydrogel with both UCST and LCST. Based on our previous publication of cross-linking-induced thermo-responsiveness (CIT) [43,44], the hydrophobic segment formed during gelation regulated the hydrophilicity of the hydrogels, and the structure formed contributed to the thermo-responsive property, as shown in Scheme 2. The large hydrophobic moiety with ADH and DTDPH cross-linking endowed the hydrogels with UCST and the PEO_23_ DNH cross-linked hydrogel became thermo-responsive with LCST. This research revealed a new type of thermo-responsive self-healing hydrogel besides PNIPAM-based hydrogels [28,37,46,52].

### 3.5. Self-Healing Property of the Hydrogels

**Figure 6:**
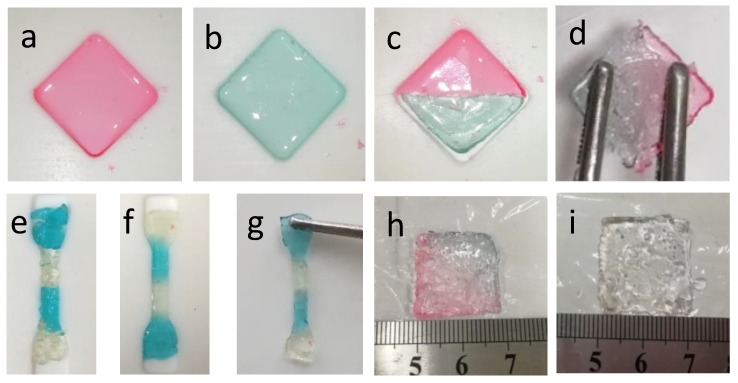
Self-healing properties of hydrogel prepared from P(AM_71_-*stat*-DAA_14_) and PEO_23_ DH cross-linking (**a**–**d**), the reshaping process (**e**–**g**), and the swell property in pH 7.4 buffer (**h**,**i**).

These interesting ketone-type acylhydrazone containing thermoresponsive hydrogels should have a self-healing property at neutral conditions without an additional stimulus [27,37,44,45]. The self-healing experiment in this study wascarried out and confirmed with a variety of methods. The diamond-shaped hydrogel plates prepared from P(AM_71_-*stat*-DAA_14_) with PEO_23_ DBH were stained into red and blue colors, respectively. Then, the two hydrogel plates were cut across the center, and the two pieces of hydrogel plates with different colors were put back into the original mould and sealed. After having contactfor 24 h, the hydrogel plate was taken out and stretched perpendicular to the cut line by tweezers. As shown in Figure 6a–d, the two hydrogel pieces merged into one whole plate and could not besplit under stretching. Additionally, the hydrogel with different colors on each side camefrom different original parts, which confirmed the self-healing property of the hydrogels (Figure 6d). However, because the pigments of red and blue are water-soluble, the color diffused across the contact line. The hydrogels were also cut into small pieces and put into a dumbbell-shaped mould with alternative colors, and after 24 h, the hydrogel was taken out to examine the self-healing result. The hydrogel became a whole bar after 24 h, and the rough surface of the hydrogel become pretty smooth through the reshaping process and couldhold its own weight under gravity, as shown in Figure 6e–g. With the same kind of dynamic bond, the hydrogel prepared from P(AM_7__2_-*stat*-DAA_28_) with PEO_23_ DNH cross-linking also self-healed in 24 h and didnot break under bending (Figure S3).

With potential application in bioscience, the dimensional stability of the hydrogel in ph 7.4 buffer is also very important. The swelling process was observed by immersing the hydrogel plate into the pH 7.4 buffer, and the plate was taken out at a predetermined time scale to measure the size change. As shown in Figure 6h,i, the side length of the hydrogel square increased from 16 mm (Figure S4) to 18 mm in the first hour, and then increased to 20 mm in 24 h. The color of the hydrogel disappeared, but the hydrogel was still one whole plated. This result showed that the hydrogel was pretty stable in pH 7.4 buffer. This property makes the self-healing hydrogel very fit for tissue repairing applications.

**Figure 7:**
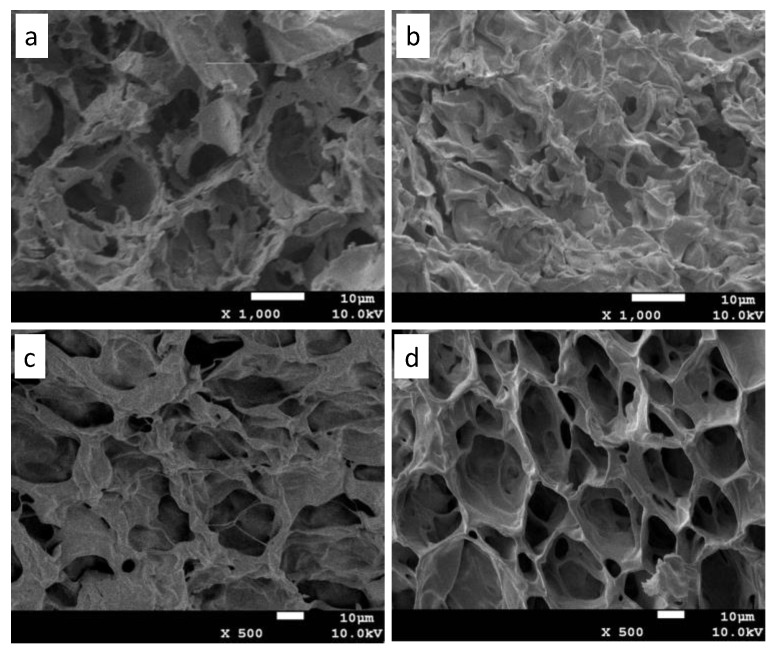
SEM images of hydrogels prepared form P(AM_71_-*stat*-DAA_14_) with (**a**) DTDPH, (**b**) ADH, (**c**) and PEO_23_ DBH cross-linking, respectively, and (**d**) P(AM_72_-*stat*-DAA_28_) with PEO_23_ DNH cross-linking.

To further investigate the structure of the hydrogels, SEM was used to observe the morphology of the hydrogels. To preserve their original structure, the hydrogels were frozen in liquid nitrogen to avoid the morphology change during freezing. The SEM images of a series of hydrogels prepared from P(AM_71_-*stat*-DAA_14_) with different cross-linkers are shown in Figure 7. The pore size of the opaque hydrogel with DTDPH cross-linking is bigger than that of ADH cross-linking (Figure 7a,b). Based on a similar cross-linking density, the hydrogel cross-linked by DTDPH was aggregated at room temperature and resulted in a larger pore size. The clear hydrogel prepared with PEO_23_ DBH also showed a large pore size, whichis because the morphology resembled during lyophilization and SEM observation based on the low *T*_g_ of PEO. At the same time, the collapsed microporous hydrogel can be figured out from the image (Figure 7c). Similar results were observed for the PEO_23_ DNH cross-linked thermo-responsive hydrogel from P(AM_72_-*stat*-DAA_28_) (Figure 7d).

### 3.6. Gel-Sol-Gel Transition of the Self-Healing Hydrogels Triggered by pH, Redox, and Group Ratios

**Figure 8:**
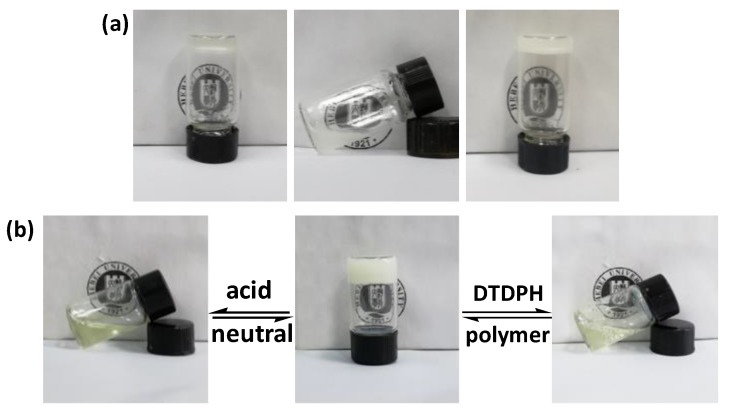
Reversible gel-sol-gel transition of hydrogels with an acylhydrazone bond. (**a**) redox of DTDPH cross-linked P(AM_77_-*stat*-DAA_8_); (**b**) pH and group ratio triggered opaque P(AM_71_-*stat*-DAA_14_) hydrogel cross-linked by DTDPH. (Credit from Hebei University).

The acylhydrazone bond is a pH sensitive bond and the disulfide bond is also a reversible bond under redox triggers [36,43]. Therefore, the hydrogels in this research also showed gel-sol-gel transition under various conditions. Besides the pH regulatingthe gel-sol-gel transition of all clear hydrogels with ADH and PEO DBH cross-linking, the clear hydrogel prepared from P(AM_77_-*stat*-DAA_8_) with DTDPH cross-linking also showed redox triggered gel-sol-gel transition, as shown in Figure 8a. When two equivalent amounts of DTT based on DTDPH wereadded into the vial, the hydrogel degraded into solution overnight; when the same equivalent of H_2_O_2_ was added, the thiol groups were oxidized into a disulfide group again and the hydrogel was regenerated.

**Scheme 3:**
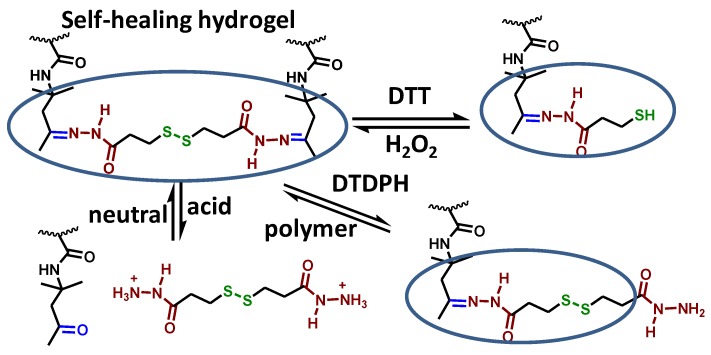
The mechanism for gel-sol-gel transition of DTDPH cross-linked P(AM_71_-*stat*-DAA_14_) hydrogel under various triggers and the comparison of the hydrophobic moiety.

The opaque hydrogel prepared from P(AM_71_-*stat*-DAA_14_) with DTDPH cross-linking also has areversible bond, but the aggregated hydrophobic domains can restrict the reversible reaction. In this research, the reversibility of this hydrogel was investigated. It is amazing to see that this hydrogel also showed gel-sol-gel transition under various conditions. When HCl was dropped onto the opaque hydrogel, the hydrogelgradually turned into clear solution in 30 min, whichis because the hydrazide group was reacted with HCl into soluble salt; when the solution was neutralized by N(C_2_H_5_)_3_, opaque hydrogel was reobtained. This hydrogel also degraded into solution by excess group ratios of DTDPH based on Carothers theory [53]. When twoamounts ofexcess DTDPH were added into 1 mL of hydrogel, the hydrogel also transformed into clear solution overnight; when the P(AM_71_-*stat*-DAA_14_) solution was added into the vial to regulate the group ratio to 1:1, hydrogel was formed again. The gel-sol-gel transitions of this hydrogel under pH and group ratios are shown in Figure 8b. More amazingly, this hydrogel also underwent redox triggered gel-sol-gel transition and the clear solution was obtained under DTT reduction (Figure S5). Based on the above result, the increased solubility of the resultant product was very important for gel-sol transition of the opaque hydrogels since our previous research revealed thatsome opaque hydrogel containing a disulfide bond cannot be cleaved by DTT [43]. The structures of resultant products are shown in Scheme 3 for comparison. Among all the above gel-sol-gel transition processes, the group ratio triggered gel-sol-gel transition does not change the pH or redox state of the hydrogels, which is more safe in bioapplications.

Based on the reversible reactivity of the opaque hyrogel prepared from P(AM_71_-*stat*-DAA_14_) with DTDPH cross-linking, this hydrogel is expected to be self-healing as well. The opaque hydrogel prepared form P(AM_71_-*stat*-DAA_14_) with DTDPH cross-linking can self-heal or heal together with clear hydrogel with ADH cross-linking (Figure S6). A possible reason for this is that the hydrophobic domain formed was not compact and couldstill perform the reversible reaction slowly. This hydrogel with a janus part is not like those hydrogels with dyeing since the different color is derived from the different phase of the hydrogel and can be maintainedseparately for a longer time. This property could have potential application to prepare thermo-responsive smart hydrogels with interesting properties, which is under research.

**Figure 9:**
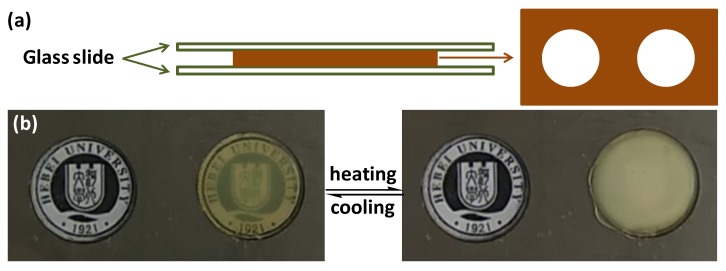
Schematic illustration (**a**) and pictures of the model thermo-switchable window at low and high temperature (**b**). (credit from Hebei University).

The thermo-responsive property of the hydrogel has various potential applications, including temperature sensors, and thermo-switchable windows in medical and commercial technologies [54]. The hydrogels prepared form P(AM_65_-stat-DAA_20_) with PEO_23_ DNH and PEO_23_ DBH cross-linking were filled in a round mould and sandwiched in two glass sheets to investigate the application of a thermo-switchable window. Figure 9 shows the scheme and the images of the process. When the hydrogels were warmed with a hair drier, the brown hydrogel turned from transparent to opaque within 40 s. When the water bag was put on the opaque hydrogel, the hydrogel become transparent gradually in about 2 min (Figure S7 and Video S1). The clear hydrogel with PEO DBH cross-linking did not show any difference in this cycle. Although the PNIPAM containing hydrogel can also show a synergistic thermo-responsive property [54], this hydrogel with cross-linking-induced thermo-responsiveness opened a new window for thermo-responsive hydrogels independent of PNIPAM.

### 3.7. Thermostability of the Copolymer and Hydrogels

**Figure 10:**
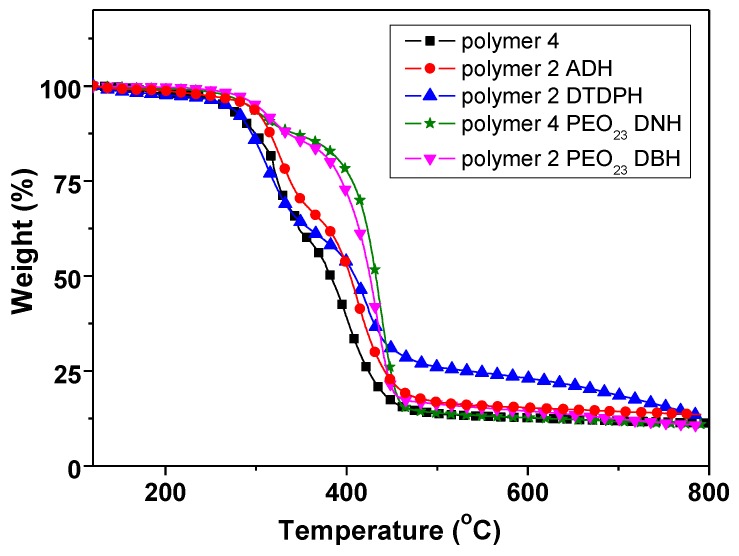
TGA curves of the polymer 4 and a series of hydrogels.

The thermogravimetricanalysis (TGA) of the polymer and the hydrogels wasdetermined to evaluate the thermal stability. As shown in Figure 10 (black line), the polymer decomposed in two steps. From 250 to 325 °C, the copolymer lost its partial weight, and other weight was lost from 325 to 475 °C. This result indicated that the side groups were cleaved from the polymer backbone. The hydrogel with ADH cross-linking also decomposed at a similar temperature range (Figure 10 red line), and at the same time, the weight loss ratio is comparable to that of the copolymer, which indicated that the attached ADH also evaporated with the cleaved side group. The hydrogel with DTDPH cross-linking showed asimilar temperature range, but the weight left ratio is much higher than other hydrogels (Figure 10 blue line). With the same reaction and dynamic covalent bond, the disulfide bond was supposed to be decomposed at a high temperature and induce side reactions. However, the hydrogels with PEO_23_ DBH and PEO_23_ DNH cross-linking also showed partial weight loss at 285 °C (Figure 10). Thisindicated that the ketone groups were partiallyconsumed and free ketone groups were left in thehydrogel [44]. This result is reasonable since the acylhydrazone bond is reversible and the steric resistanceof the ketone group is high. As a result, a higher DAA composition is preferred and a copolymer with a low DAA composition always results in a low cross-linking density.

## 4. Conclusions

In summary, thermo-responsive self-healing hydrogels with both UCST and LCST were prepared from P(AM-*stat*-DAA) with diacylhydrazide cross-linkers through the CIT mechanism. The thermo-response of the hydrogels wasdependent on the resultant hydrophobic moiety formed during cross-linking. With a large hydrophobic moiety with ADH and DTDPH cross-linking, hydrogels showed UCST, while LCST around body temperature wasgenerated by PEO_23_ DNH cross-linking. The results also showed that the hydrogels self-healed without any interference of a catalyst or triggers. The hydrogels also showed that the pH, group ratio, and redox triggered gel-sol-gel transitions because a variety of reversible bonds existed in the hydrogels. The hydrogels with a CIT property have great potential applicationsin temperature-sensitive drug delivery or thermo-switchable electronicdevices.

## Figures and Tables

**Table 1:** Copolymers of P(AM-*stat*-DAA)with different compositions.

Copolymer	Composition	DAA Molar Ratio ^a^	*M*_n_ (kg mol^−1^) ^b^	*T*_g_^c^
P1	P(AM_77_-*stat*-DAA_8_)	9.4	6.8	188.8
P2	P(AM_71_-*stat*-DAA_14_)	16.5	7.4	180.5
P3	P(AM_65_-*stat*-DAA_20_)	23.2	8.0	174.6
P4	P(AM_72_-*stat*-DAA_28_)	28.0	9.9	159.3

^a^ Calculated from ^1^H NMR spectroscopy. ^b^ Evaluated by monomer conversion. ^c^ Characterized by DSC.

## Supplementary Materials

The following are available online at https://www.mdpi.com/2073-4360/11/3/490/s1.
