# Supplementary material for: Self-Healing Hydrogels with both LCST and UCST through Cross-Linking Induced Thermo-Response
Source: Polymers (Basel). 2019 Mar 13;11(3):490. doi: 10.3390/polym11030490 (PMC6473816; doi:10.3390/polym11030490)
Supplement: Supplementary file 1 [file polymers-11-00490-s001.zip › Supplementary Materials polymers.doc]

Supplementary Materials:

Self-Healing Hydrogels with both LCST and UCST through Cross-Linking Induced Thermo-Response

Haifeng Zhao^1#^, Heng An^2#^, Baozhong Xi^1^, Yan Yang^1^, Jianglei Qin^2^,Yong Wang^3^, Yingna He^1*^ and Xinguo Wang^4^*

^1^	Hebei Key Laboratory of Chinese Medicine Research on Cardio-Cerebrovascular Disease, Pharmaceutical College, Hebei University of Chinese Medicine, Shijiazhuang City, Hebei Province 050200, China; Zhaohf226@126.com(H. Z.); (B. X.); 15176821860@163.com(Y. Y.) ;heyingna2002@163.com(Y. H.)

_2_	College of Chemistry and Environmental Science, Hebei University, 180 East Wusi Road, Baoding City 071002, China; flyingstock@foxmail.com (H. A.); qinhbu@iccas.ac.cn(J. Q.)

^3^ Medical College, Hebei University, Baoding City 071002, China. ywangnk@163.com (Y. W.)

^4^ Hebei TCM Formula Granule Technology Innovation Center & TCM Formula Granule Research Center Of Hebei Province University, Hebei University of Chinese Medicine, Shijiazhuang City, Hebei Province 050200, China wangxinguozy@163.com

\# Haifeng Zhao and Heng An contributed equally to this work.

* Correspondence: Y. H and X. W; heyingna2002@163.com; Tel.: +86 0311 8992 6307

Contents

**Scheme S1** Chemical structure of ADH, DTDPH, PEO_23_ DBH, PEO_23_ DNH.

**Figure S1** DSC curves of P(AM-*stat*-DAA).

**Figure S2** Reversible curve of PEO_23_ DNH cross-linked P(AM_72_-*stat*-DAA_28_).

**Figure S3** The hydrogel prepared from P(AM_72_-*stat*-DAA_28_) with PEO_23_ DNH cross-linking self-healed in 24 h.

**Figure S4** Size of the hydrogel plate before swelling experiment.

**Figure S5** Opaque hydrogel prepared form P(AM_71_-*stat*-DAA_14_) with DTDPH cross-linking degraded by excess of DTDPH.

**Figure S6** The self-healing of clear hydrogel and opaque hydrogel prepared from P(AM_71_-*stat*-DAA_14_) with ADH and DTDPH cross-linking healed into a whole plate in 24 h.

**Figure S7** The images of the hydrogel prepared from P(AM_72_-*stat*-DAA_28_) cooled under cooled water (left: PEO_23_ DBH cross-linking; right PEO_23_ DNH cross-linking).

Scheme S1 Chemical structure of ADH, DTDPH, PEO_23_ DBH, PEO_23_ DNH.

**Figure S1.** DSC curves of P(AM-*stat*-DAA).

**Figure S2.** Reversible curve of PEO_23_ DNH cross-linked P(AM_65_-*stat*-DAA_20_).

**Figure S3.** The hydrogel prepared from P(AM_72_-*stat*-DAA_28_) with PEO_23_ DNH cross-linking self-healed in 24 h.

**Figure S4.** Size of the hydrogel plate before swelling experiment.

**Figure S5.** Opaque hydrogel prepared form P(AM_71_-*stat*-DAA_14_) with DTDPH cross-linking degraded by excess of DTDPH.

**Figure S6.** The self-healing of clear hydrogel and opaque hydrogel prepared from P(AM_71_-*stat*-DAA_14_) with ADH and DTDPH cross-linking healed into a whole plate in 24 h.

**Figure S7.** The images of the hydrogel prepared from P(AM_72_-*stat*-DAA_28_) cooled under cooled water (left: PEO_23_ DBH cross-linking; right PEO_23_ DNH cross-linking).
